# Reoperation rate and implants’ position variation features of displaced femoral neck fractures with sliding compression or length-stable fixation in young and middle-aged population

**DOI:** 10.1186/s12891-022-05956-9

**Published:** 2022-11-18

**Authors:** Xiao-zhong Zhu, Wei Wang, Sheng-hui Wu, Jiong Mei

**Affiliations:** 1 Department of Orthopaedic Surgery, Shanghai Sixth People’s Hospital Affiliated to Shanghai Jiaotong University School of Medicine, Shanghai, 200233 China; 2 Department of Biomedical Engineering, The Hong Kong Polytechnic University, Hong Kong, China

**Keywords:** Femoral neck fracture, Cannulated screw, Length-stable fixation, Sliding compression fixation, Reoperation, Spatial variation

## Abstract

**Introduction:**

Sliding compression fixation and length-stable fixation are two basic internal fixation concepts in the treatment of displaced femoral neck fractures. In this study, we aimed to compare the reoperation rates for different methodologies of internal fixation for femoral neck fractures in young and middle-aged population.

**Materials and methods:**

This a retrospective study. A total of 215 patients with displaced femoral neck fractures treated with cannulated screw fixation were enrolled and divided into the sliding compression and length-stable groups according to the fixation pattern. The occurrence of and reason for revision surgery within one year were recorded. Forty-five patients with complete CT data (including CT scanning on the first postoperative day and at the last follow up) were selected from the total sample. A newly established computerized image processing method was used to evaluate variations in the spatial location of screws.

**Results:**

The reoperation rate was significantly higher in the length-stable group (23.8%) than in the sliding compression group (7.3%). The rate of revision surgery due to nonunion was also higher in the length-stable group (11.4%) than in the sliding compression group (1.8%). However, no significant difference was observed in terms of joint penetration or soft tissue irritation. The sliding compression group (6.58 ± 3.18 mm) showed higher femoral neck shortening than length-stable group (4.16 ± 3.65 mm). When analyzing the spatial variations, a significantly greater screw withdrawal distance was observed in the sliding compression group than in the length-stable group, but with a smaller rotation angle.

**Conclusion:**

Length-stable internal fixation of displaced femoral neck fractures may lead to an increased reoperation rate in young and middle-aged population.

**Trial registration:**

Name of the registry: Chinese Clinical Trial Registry.

Trial registration number: ChiCTR2000032327.

Trial registration date: 2020–4-26.

## Introduction

Sliding compression fixation and length-stable fixation are the two main methods currently used in the treatment of femoral neck fractures [1]. Parallel fixation with partially threaded cannulated screws (PTCSs) is the most commonly used fixation method in clinical practice. In the past fifty years, researchers have made many improvements to internal fixation methods. Some scholars have also proposed length-stable fixation because of the impairment of hip joint function brought about by femoral neck shortening after sliding compression fixation [2, 3]. Researchers have also proposed fixation devices such as fully threaded cannulated screws (FTCSs) and proximal femur locking plates (PFLPs) [4, 5]. However, many patients require reoperation within one year after the first operation, and the reoperation rate is 10–15% [6–10] even among patients with stable fractures without displacement or valgus-impacted fractures. To date, there is no consensus in the medical community on the most appropriate technique for fixing femoral neck fractures [11]. Do different methodologies of internal fixation for femoral neck fractures affect the reoperation rate? Based on this question, we divided patients treated with cannulated screw fixation into the length-stable and sliding compression fixation groups according to the threads and orientation of the applied screws. The aim of this study is to compare the early reoperation rates for different methodologies of internal fixation for FNFs in young and middle-aged population. We also established a new computerized image processing method to model the implants in three dimensions and then analyzed variations in the spatial location of the implants.

## Materials and methods

This is a retrospective study approved by the institutional ethics committee and performed in line with the principles of the Declaration of Helsinki. Informed consent was obtained from all individual participants included in the study.

Patients with displaced femoral neck fractures admitted to our hospital from January 2018 to August 2020 who underwent fixation with cannulated screws were included. The inclusion criteria were as follows: (1) young and middle-aged population (age between 20 and 65 years), with a history of traumatic injury; (2) diagnosis of displaced femoral neck fracture (Garden type III to IV); and (3) satisfactory reduction achieved by treatment with cannulated screw fixation. The reduction effect was evaluated by Garden’s alignment index [12, 13] combined with Lowell’s curve; the angle between the medial trabecular stream in the femoral head and the medial cortex of the femoral shaft (trabecular-shaft angle) should be larger than 155° on postoperative anteroposterior radiographs of the hip and smaller than 180° on lateral radiographs [12]. The concave outline of the femoral neck should meet the convex outline of the femoral head in an S-shaped curve superiorly, inferiorly, anteriorly and posteriorly [14]. All included patients completed follow-up for at least 1 year after surgery. The exclusion criteria were as follows: (1) age less than 20 years or more than 65 years; (2) patients without a native contralateral hip; (3) treatment with other implants or hip joint arthroplasty; and (4) failure to achieve satisfactory reduction or application of open reduction; (5) Follow-up less than 12 months (unless treatment failed); (6) accompanied acetabular, peritrochanteric or femoral head fractures or hip dislocations.

### Sample size calculation and grouping

**Fig. 1:**
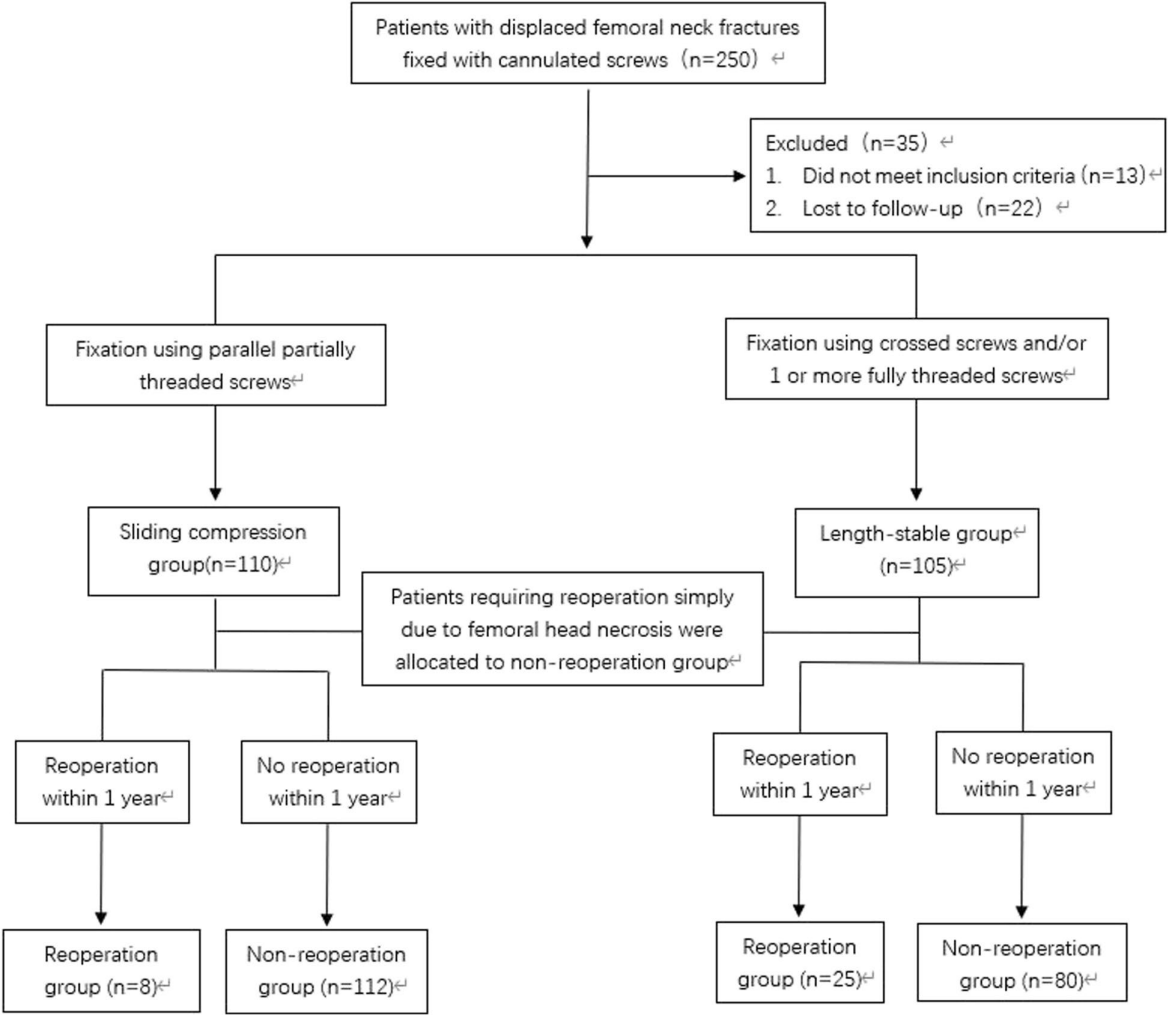
Study cohort recruitment flowchart

The patients were divided into the sliding compression and length-stable fixation groups according to the threads and orientation of the applied cannulated screws. Patients were included in the length-stable group when one or more FTCSs were used or when the screws were cross-fixed. Patients were included in the sliding compression group only when all screws were partially threaded and parallel to each other. The sample size calculation was based on a type I error (α) of 0.05 and power of 80% (1-β), based on data obtained from previous studies and our preliminary research. Ninety-seven patients were required for each group, and we finally set the sample size to 125 considering a possible dropout rate of 20%. All patients were required to be followed up for at least 12 months. The occurrence of and reason for reoperation were recorded. The patients who undergone reoperation simply due to femoral head necrosis were allocated to the non-reoperation group (Fig. 1).

### Variations in spatial location of implants

**Fig. 2:**
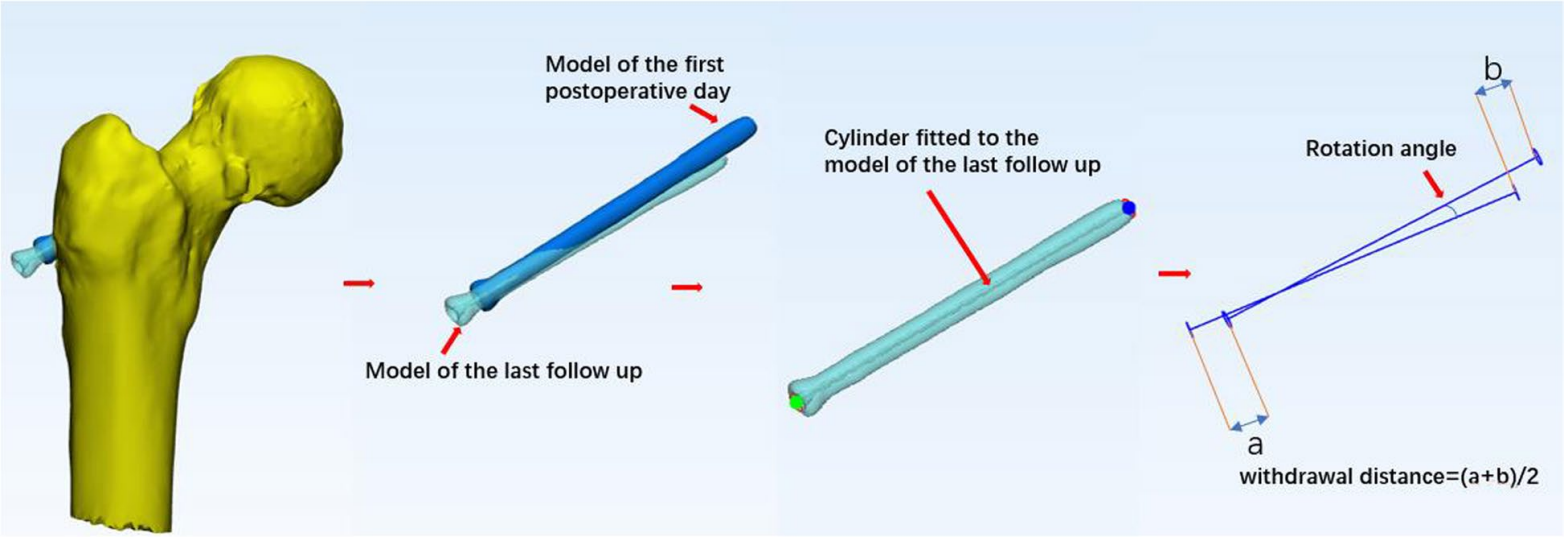
Cannulated screws modeled using data from different time points were aligned based on the femoral shaft in 3-matic software; then, a cylinder was fitted to each screw, and the corresponding axis was displayed. The screw withdrawal distance and screw rotation angle were measured to analyze variations in the spatial location of the implants

To evaluate differences of the spatial variations of the implants between the two groups, we screened 45 patients from the total sample who had complete CT data(At our institution, X-rays are usually obtained on the first day after surgery; CT scanning is seldom performed but is necessary to obtain the following measurements).CT images obtained on the first postoperative day and at the last follow-up (the last follow-up for patients without reoperation or the last follow-up before reoperation) were imported into Mimics software in DICOM format. Each cannulated screw was modeled separately, and the models were then imported into 3-matic software in STL format. The models of data from different time points were aligned based on the femoral shaft using the N-points Registration and Global Registration tools. Then, a cylinder was fitted to each cannulated screw, and the corresponding axis was displayed. The mean of proximal and distal displacement of each cannulated screw was used to describe the screw withdrawal distance. The angle between the axis of the two cylinders from the different time points was used to describe the screw rotation angle (Fig. 2).

### Statistical analysis

**Fig. 3:**
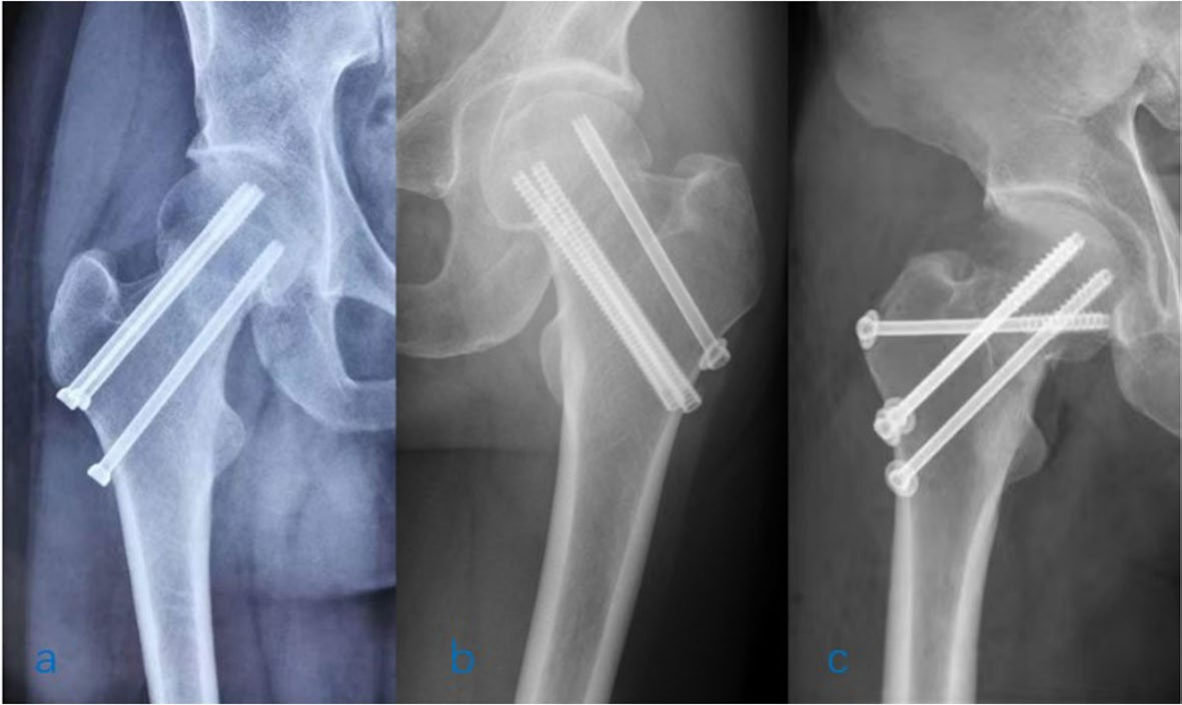
**a** Fixation with three parallel PTCSs. This case was included in the sliding compression group, and all three screws were considered sliding compression screws. **b** Fixation with three parallel screws, one partially threaded screw and two fully threaded screws. This case was included in the length-stable group, and all three screws were considered length-stable screws, including the lag screw. **c** Fixation with four crossed partially threaded screws. This case was included in the length-stable group, and all screws were considered length-stable screws

Data regarding reoperation in the sliding compression and length-stable groups were recorded separately, along with general demographic information, such as age and sex. Fracture displacement and Pauwels angle were evaluated by physicians with more than 5 years of experience of orthopaedics trauma; disagreements were adjudicated by a senior specialist. The cannulated screws used in the treatment of the 45 patients mentioned in the former paragraph were divided into length-stable screws and sliding compression screws. As opposed to sliding compression screws in the general sense, only screws applied in the sliding compression group were defined as sliding compression screws, while both partially threaded screws and fully threaded screws applied in the length-stable group were defined as length-stable screws (Fig. 3). Categorical data are presented as numbers and/or percentages. Continuous variables are presented as the mean ± standard deviation. Differences among groups were assessed using Pearson’s chi-squared test or one-way ANOVA, unless indicated otherwise. Confidence intervals were set at 95%, and all data were analyzed using SPSS software.

## Results

A total of 215 patients were included in the study; 110 (51.2%) were treated with sliding compression fixation, and 105 (48.8%) were treated with length-stable fixation. The general demographic information and reoperation rates of patients in the two groups are presented in Table 1. There was no significant difference between the two groups in terms of age and Pauwels angle, but the proportion of male patients was higher in the length-stable fixation group than in the sliding compression fixation group (*p* < 0.05). A significantly higher reoperation rate was observed in the length-stable group (25 out of 105, 23.8%) than in the sliding compression group (8 out of 110, 7.3%) (***p* = 0.001**). The sliding compression group (6.58 ± 3.18 mm) showed higher femoral neck shortening than length-stable group (4.16 ± 3.65 mm) (***p* = 0.022**). In the sliding compression group, significantly fewer patients required reoperation due to nonunion than in the length-stable group (***p* = 0.004**). No difference was found in reoperation rates between the two groups in terms of soft tissue irritation or joint penetration.

**Table 1:** The general demographic information and reoperation rate of the two groups

	Sliding compression fixation (*n* = 110)	Length-stable fixation (*n* = 105)	***P* value**
Age (yr)	53.75 ± 11.94^a^	51.23 ± 11.81	0.121
Gender			
Male	45(40.9)^∗^	71(67.6)	**0.000**
Female	65(59.1)	34(32.4)	
Pauwels > 50	70(63.6)	72(68.6)	0.445
Reoperation	8(7.3)	25(23.8)	**0.001**
Non-union	2(1.8)	12(11.4)	**0.004**
Joint penetration	4(3.6)	10(9.5)	0.080
Soft tissue irritation	2(1.8)	3(2.9)	0.613
Femoral neck shortening (mm)	6.58 ± 3.18	4.16 ± 3.65	**0.022**

^*^Data are presented as the number of patients, with the percentage in parentheses, unless otherwise noted. ^a^Standard deviation

Of the 215 patients mentioned above, a total of 45 patients had complete follow-up CT data, which were collected in DICOM format for computerized modeling. Of them, 23 were in the sliding compression group, and 22 were in the length-stable group. According to the method of screw grouping applied in this study, there were 80 length-stable screws and 82 sliding compression screws in the 45 patients with complete CT data. By comparing the screw withdrawal distance and screw rotation angle determined by the CT on the first postoperative day and at the last follow-up(for those who didn’t receive reoperation)/before reoperation (for those who did receive reoperation) between the two groups, it was found that the mean screw withdrawal distance was significantly shorter in the length-stable group (2.77 ± 2.34 mm) than in the sliding compression group (3.75 ± 3.49 mm) (***p* = 0.036)**, while the screw rotation angle was significantly greater in the length-stable group (2.02 ± 1.92°) than in the sliding compression group (1.47 ± 1.46°) (***p* = 0.042**) (Table 2).

**Table 2:** Variations in the spatial location of screws

	sliding compression screws (*n* = 82)	length-stable screws(*n* = 80)	***P* value**
screw withdrawal distance(mm)	3.75 ± 3.49	2.77 ± 2.34	**0.036**
rotation angle (°)	1.47 ± 1.46	2.02 ± 1.92	**0.042**

**Fig. 4:**
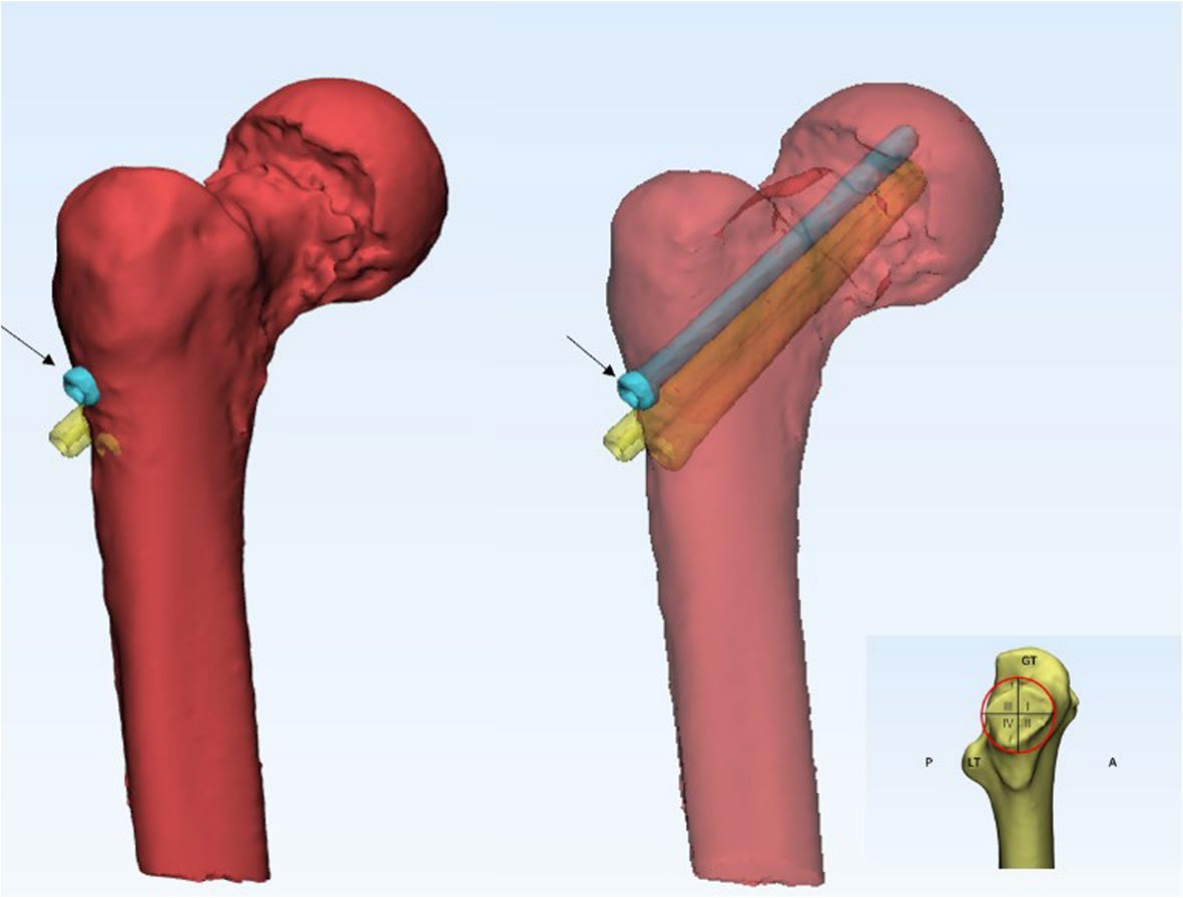
Semitransparent examination of the femur showing localization of the screw with the longest withdrawal distance (arrow) and the bone defect in the same quadrant

**Fig. 5:**
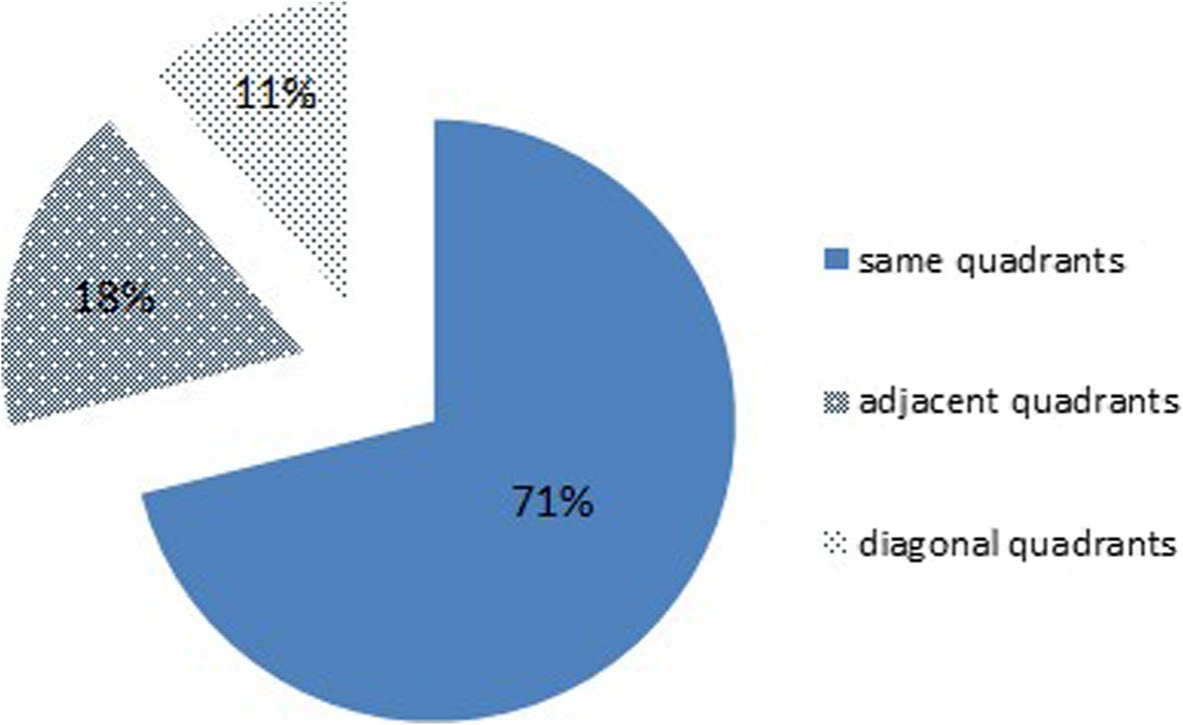
Positional relationship between the screw with the longest withdrawal distance and the bone defect

There was also an unexpected finding on further analysis of the CT images obtained on the first day after the operation in 45 patients in this study. The distal surface of the fracture end was first divided into four quadrants using two lines perpendicular to each other: the anterosuperior quadrant was the first quadrant; the anteroinferior quadrant was the second quadrant; the posterosuperior quadrant was the third quadrant; and the posteroinferior quadrant was the fourth quadrant. We recorded the quadrant containing the screw with the longest withdrawal distance and the major quadrant of the fracture defect after fracture reduction (Fig. 4). We found that these quadrants were the same in 71% of the cases (32 out of 45), adjacent in 18% of cases (8 out of 45), and diagonal from each other in 11% of cases (32 out of 45) (Fig. 5).

## Discussion

In the treatment of femoral neck fractures, fracture union is the common goal of both sliding compression and length-stable fixation. Sliding compression prioritizes union at the expense of uncontrolled shortening [15, 16]. But in contrast, as the result shown in this study, length-stable fixation may compensate the former‘s shortages at the expense of higher reoperation rate.

Controllable shortening of the femoral neck (up to 10 mm) is generally harmless and may occur as early as 1 week postoperatively in some cases [16]. Generally, screw withdrawal does not occur in cases of valgus-impacted or nondisplaced femoral neck fractures (Garden types I and II), whereas almost all displaced femoral neck fractures treated with sliding compression fixation experience screw withdrawal on average of 10 mm [17], with potentially more in comminuted patterns. Unless the screw withdrawal is associated with fracture displacement, particularly varus, apex anterior deformity or rotational malalignment, it does not typically require surgical intervention [18].

The importance of restoring the length of the femoral neck in terms of the functional prognosis has been highlighted in a number of publications. Zlowodzki et al. retrospectively evaluated the effect of femoral neck shortening on hip function and suggested that femoral neck shortening has a negative impact on daily function [2, 3, 19]. There were statistically significant differences between patients with no/mild shortening (< 5 mm) and patients with severe shortening (> 10 mm) in SF-36 physical functioning scores [3]. Therefore, a number of authors have advocated for the use of length-stable fixation for femoral neck fractures. FTCSs are increasingly being used for the internal fixation of femoral neck fractures. Biomechanical studies have shown that in femoral neck fractures, FTCSs have the advantage of resisting shear stress [10] and bending force and are less prone to fixation failure [20, 21]. However, when performing length-stable internal fixation, if the contacting portions of the fracture ends after reduction do not coincide with the compressed or defect area, instability may occur. Essentially, this outcome is similar to failure to achieve anatomical fracture reduction. Furthermore, if stability of the fracture ends is not ensured, nonunion may occur, leading to potential for screw penetration into the joint and ultimately internal fixation failure [22, 23]. Thus, it is undesirable to pursue maintenance of the femoral neck length and to ignore the bone defect and fracture end stability [24].

All 162 cannulated screws in this study showed varying degrees of displacement. By comparing the screw withdrawal distance and rotation angle between the sliding compression and length-stable groups during fracture healing, we found that the screw withdrawal distance (***p*** = **0.036**) and femoral neck shortening (***p* = 0.022)** was greater in the sliding compression group than in the length-stable group, while the rotation angle was smaller in the sliding compression group than in the length-stable group (***p* = 0.042**). In other words, the sliding compression screws moved mainly axially along the femoral neck axis in terms of spatial position, whereas the movement of the length-stable was dominated by rotation. One reason for this maybe that the movement in a length stable construct gives rise to differential screw toggling which encourages a rotational movement. In terms of the main causes for reoperation, most of the patients (4 out of 8) in the sliding compression group underwent implant removal due to discomfort and skin irritation caused by screw withdrawal. However, many patients did not want to undergo another operation, despite notable screw withdrawal or femoral neck shortening on postoperative radiography. Many of these patients even reported no significant discomfort. Previous literature reported that gait pattern change may compensate for the hip joint functional limitation [25]. While the variation of the spatial location of screws in the length-stable group suggests fracture end instability and femoral head rotation. Therefore, when analyzing the reasons for reoperation, we also found that the proportion of patients who underwent reoperation for nonunion (12 out of 25) was greater in this group than in the sliding compression group [26]. Such complication is unbearable for patients. The difference between the composition of the chief complaints for revision surgery may explain why the reoperation rate was much lower in the sliding compression group.

In terms of Pauwels angle, no significant difference was observed between length-stable group and sliding compression group (Table 1). Shear stress and varus force increase along more vertically oriented fractures, resulting in higher risk of fracture displacement and ultimately nonunion [27]. Contact surface of fracture end play an important role in fracture union. Of the 33 patients who received revision surgery, 20 fractures showed Pauwels angle larger than 50°.

For the phenomenon that the major quadrant of the fracture defect is essentially in agreement with the quadrant containing the screw with the longest withdrawal distance, we speculate that the variation in the spatial location of cannulated screws is highly correlated with the location of the bone defect in femoral neck fractures, particularly after reduction. In fact, a long time ago, when Kauffman et al. compared the biomechanical stability of femoral neck fractures fixed with three parallel screws in inverted triangles with that of fractures fixed with four parallel screws in a diamond shape, they found no significant difference when posterior bone defects were not observed [28]. If there was a posterior cortical defect, better stability was obtained with 4 screws. It is evident that the addition of screws on the side of the defect enhances the stability of the fracture. The results of this study support our speculation. Of course, in this study, the surface of the fracture end was simply divided into four quadrants, so the distance between the cannulated screws and the center of the bone defect was not quantified. Additionally, the rotation angle was not taken into consideration, which may have also contributed to slight bias in the results. Therefore, more comprehensive and detailed experiments and analyses are needed to confirm this conjecture.

There are several other limitations to this study. Since this was a retrospective study, there was no intervention regarding the sex ratio in the two groups. Therefore, there was a higher percentage of males in the length-stable group in this study. Perhaps the male patients had higher energy trauma which resulted in significant comminution and the surgeon's preference for length stable constructs for more reliable stability. Previous study also reported that compared with patients with nondisplaced fractures, those with displaced injuries were more frequently male, experienced different fracture pattern, were more often treated with a fixed-angle device [29]. Second, when analyzing the reasons for reoperation, due to sample size limitations, we could only conclude that the proportion of patients who underwent reoperation for nonunion was greater in the length-stable group than in the sliding compression group. The other two negative results (for soft tissue irritation due to screw withdrawal and joint penetration) require further verification due to the limited sample size. Finally, the minimum follow-up for inclusion in this study was limited to only 12 months. Although short-term follow-up may be able to analyze fracture healing and stability of the implants, it limits the analysis of osteonecrosis, which may take 2 or more years to manifest.

## Conclusion

In conclusion, the consequences of screw withdrawal caused by sliding compression and fracture end instability are different. The variation in the spatial location of sliding compression screws appears to be featured by axial motion, which facilitates union of the fracture ends and promotes fracture healing. While the motion mode of length-stable screws seems be mainly featured by rotational motion, suggesting fracture end instability, which may eventually lead to serious complications such as nonunion and greatly increase the possibility of reoperation. Therefore, in clinical practice, we recommend parallel fixation with PTCSs for displaced femoral neck fractures to reduce the reoperation rate in young and middle-aged population.

## Data Availability

Data will be available from the corresponding author on rationale request. Confidential patient data should not be shared.
